# Chronic Stress Alters Synaptic Inhibition/Excitation Balance of Pyramidal Neurons But Not PV Interneurons in the Infralimbic and Prelimbic Cortices of C57BL/6J Mice

**DOI:** 10.1523/ENEURO.0053-24.2024

**Published:** 2024-08-23

**Authors:** Diana Rodrigues, Cátia Santa, Bruno Manadas, Patrícia Monteiro

**Affiliations:** ^1^Life and Health Sciences Research Institute (ICVS), School of Medicine, University of Minho, Braga 4710-057, Portugal; ^2^ICVS/3B’s-PT Government Associate Laboratory, Braga/Guimaraes, Braga 4710-057, Portugal; ^3^Biomedizinisches Centrum München (BMC), Ludwig-Maximilians-Universität München, Munich 82152, Bayern, Germany; ^4^CNC-Center for Neuroscience and Cell Biology, University of Coimbra, Coimbra 3004-517, Portugal; ^5^Centre for Innovative Biomedicine and Biotechnology (CIBB), University of Coimbra, Coimbra 3004-517, Portugal; ^6^Department of Biomedicine – Experimental Biology Unit, Faculty of Medicine, University of Porto, Porto 4200-319, Portugal; ^7^RISE-Health, Health Research Network, Porto 4200-319, Portugal

**Keywords:** chronic stress, electrophysiology, I/E ratio, mPFC, neuroproteomics

## Abstract

The medial prefrontal cortex (mPFC) plays a pivotal role in regulating working memory, executive function, and self-regulatory behaviors. Dysfunction in the mPFC circuits is a characteristic feature of several neuropsychiatric disorders including schizophrenia, depression, and post-traumatic stress disorder. Chronic stress (CS) is widely recognized as a major triggering factor for the onset of these disorders. Although evidence suggests synaptic dysfunction in mPFC circuits following CS exposure, it remains unclear how different neuronal populations in the infralimbic (IL) and prelimbic (PL) cortices are affected in terms of synaptic inhibition/excitation balance (*I*/*E* ratio). Here, using neuroproteomic analysis and whole-cell patch-clamp recordings in pyramidal neurons (PNs) and parvalbumin (PV) interneurons within the PL and IL cortices, we examined the synaptic changes after 21 d of chronic unpredictable stress, in male mice. Our results reveal distinct impacts of CS on PL and IL PNs, resulting in an increased *I*/*E* ratio in both subregions but through different mechanisms: CS increases inhibitory synaptic drive in the PL while decreasing excitatory synaptic drive in the IL. Notably, the *I*/*E* ratio and excitatory and inhibitory synaptic drive of PV interneurons remained unaffected in both PL and IL circuits following CS exposure. These findings offer novel mechanistic insights into the influence of CS on mPFC circuits and support the hypothesis of stress-induced mPFC hypofunction.

## Significance Statement

In unveiling distinct impacts of chronic stress (CS) on synaptic inhibition–excitation balance (*I*/*E* ratio) within the medial prefrontal cortex's infralimbic and prelimbic subregions, this study not only deepens our understanding of the intricate neurobiological responses to stress but also highlights a crucial factor in the pathophysiology of neuropsychiatric disorders. The differential modulation of *I*/*E* ratio in pyramidal neurons, coupled with the resilience of parvalbumin interneurons to CS within these subregions, underscores a nuanced susceptibility of prefrontal circuits. These findings contribute vital mechanistic insights into stress-related neuropsychiatric disorders. Moreover, we are releasing a comprehensive proteomic dataset to the research community, providing a valuable resource for future studies to explore the molecular underpinnings of stress and its effects on neural circuits.

## Introduction

The medial prefrontal cortex (mPFC) serves as a pivotal hub in the regulation of working memory, executive function, and self-regulatory behaviors (Miller, 2000; Clark et al., 2004; Lara and Wallis, 2015). Dysregulation of mPFC activity has been implicated in various stress-related disorders, including depression (Murray et al., 2011; McEwen et al., 2012), anxiety (Park and Moghaddam, 2017), and post-traumatic stress disorder (Koenigs and Grafman, 2009). The susceptibility of the mPFC to the deleterious effects of chronic stress (CS) is well established (Arnsten, 2009; Moghaddam, 2016), and emerging evidence suggests that CS-induced prefrontal hypofunction may play a pivotal role in the etiology of these disorders.

In rodent models, CS has been shown to impair mPFC-mediated executive functions, including spatial reference and working memory (Mizoguchi et al., 2000), behavior flexibility (Liston et al., 2006; Cerqueira et al., 2007, 2005), and decision-making (Dias-Ferreira et al., 2009; Graybeal et al., 2012). Accordingly, individuals affected by stress-related disorders not only exhibit neuronal atrophy, decreased volume, and altered connectivity of the dorsolateral cortex (the primate functional and neuroanatomical homolog of the rodent mPFC (Drevets et al., 2008) but also manifest deficits in these crucial behavioral domains (Schwabe and Wolf, 2009, 2010; Guenzel et al., 2014).

The infralimbic (IL) and prelimbic (PL) cortices, the two major subregions within the rodent mPFC, are recognized for their presumed opposing roles in orchestrating control over executive behaviors (Hok et al., 2005; Tanji and Hoshi, 2008; Sierra-Mercado et al., 2011; Giustino and Maren, 2015; Ito et al., 2015). Comprising medial–lateral layers, both subregions house a population of glutamatergic pyramidal neurons (PNs), whose activity is intricately regulated by local GABAergic interneurons. Prior investigations into the impact of CS on mPFC function primarily focused on PNs, particularly regarding CS-induced structural remodeling. These investigations revealed dendritic atrophy and decreased spine density in PNs within both IL and PL cortices, hinting at disruption in excitatory glutamatergic transmission after CS exposure (Seib and Wellman, 2003; Cook and Wellman, 2004; Radley et al., 2004; Radley and Morrison, 2005; Dias-Ferreira et al., 2009). Nevertheless, recent evidence has broadened our understanding by uncovering dysfunction in inhibitory GABAergic transmission. Notably, dendritic hypertrophy has been observed in a specific subset of cortical parvalbumin (PV) interneurons, the Martinotti cells (Gilabert-Juan et al., 2013). Additionally, alterations in the levels of various genes and proteins involved in GABAergic signaling have also been documented (Gilabert-Juan et al., 2013; Ma et al., 2016; Shepard et al., 2016; Banasr et al., 2017). The collective findings from these studies raise the intriguing possibility of an altered inhibition/excitation balance (*I*/*E* ratio) in stress-related disorders, potentially leading to prefrontal hypofunction (Luscher and Fuchs, 2015; Fogaça and Duman, 2019; Zhang et al., 2021).

Here, by performing cell-type-specific patch-clamp recordings, we directly assessed the *I*/*E* ratio, revealing distinct impacts of CS on both PL and IL PNs. An increased *I*/*E* ratio was observed in both subregions, albeit through different mechanisms. Specifically, in the PL region, CS increased GABAergic synaptic transmission, leading to an elevated inhibitory synaptic drive in PNs. In contrast, in the IL region, CS decreased glutamatergic synaptic transmission, resulting in reduced excitatory synaptic drive in PNs. Notably, the *I*/*E* ratio and both excitatory and inhibitory synaptic drives of PV interneurons remained unaffected in both PL and IL circuits following CS exposure. These findings provide novel mechanistic insights into the influence of CS on prefrontal cortex circuits and lend support to the hypothesis that an excitatory/inhibitory imbalance may serve as a potential mechanism by which CS induces dysfunction in the mPFC.

## Materials and Methods

### Animals

All experiments were done in accordance with European Community Council Directives (2010/63/EU) and the Portuguese law DL No. 113/2013 for the care and use of laboratory animals. All animal procedures were approved by local authorities Direção Geral de Alimentação e Veterinária (ID: DGAV 8519) and the Ethics Subcommittee for the Life Sciences and Health (SECVS) of the University of Minho (ID: SECVS 01/18). Mice were housed at constant temperature (22°C) and humidity (55%), under standard 12/12 h light/dark cycle (lights on from 8 A.M. to 8 P.M.) with *ad libitum* access to food (4RF21, Mucedola) and water. Only male mice were used in this study. C57BL/6 mice were used for proteomic analysis and whole-cell patch-clamp recordings from pyramidal cells. Pvalb-tdTomato (JAX #027395; Kaiser et al., 2016) mice were used for whole-cell patch-clamp recordings from PV interneurons. All mice used were bred on a pure C57BL/6 background, and littermates were randomly assigned to the control or CS group. Mice were housed separately by the experimental group. Primers used for genotyping: Pvalb-tdTomato (5′-ACT GCA GCG CTG GTC ATA TGA GC-3′ and 5′-ACT CTT TGA TGA CCT CCT CG-3′).

### Animals

Five-week-old male littermates were randomly assigned to two different experimental groups and were either left undisturbed in their home cages (control group) or exposed to a stress protocol (CS group), as previously described (Rodrigues et al., 2022; Rodrigues and Monteiro, 2023). Briefly, the stress protocol consisted in exposing the animals once a day, throughout 21 d, to one of three stressors: forced swimming, restraint, or social defeat. To monitor the efficacy of the CS protocol, weekly body weights and *postmortem* thymus and adrenal gland weights were measured.

### Proteomic sample preparation

Synaptosomal samples for proteomic analysis were prepared from the prefrontal cortex of control and stressed mice. Briefly, mice were anesthetized with avertin (tribromoethanol 20 mg/ml; dose of 0.5 mg/g body weight) and decapitated, and the prefrontal cortex was microdissected in ice and snap-frozen on liquid nitrogen. Prefrontal cortex tissue from three mice (∼60 mg) was pooled together to generate one sample. Synaptosomal fractions were obtained by homogenizing the tissue in 3 ml ice-cold HEPES buffer (4 mM HEPES, pH 7.4, 0.32 M sucrose) at 4°C, using a mechanical tissue grinder (30–40 strokes at 900 rpm). Homogenates were centrifuged for 15 min at 900 × *g* at 4°C, and supernatants were subsequently centrifuged for 15 min at 900 × *g* at 4°C. The supernatants were then centrifuged at 18,000 × g for 15 min, and the pellet was resuspended with 1.5 ml of HEPES buffer and centrifuged at 18,000 × g for 15 min at 4°C. The resulting pellet was dissolved in 3 ml of hypo-osmotic buffer (4 mM HEPES, pH 7.4), and eight manual strokes were applied. Hypo-osmotic synaptosomal fractions were rotated for 1 h at 4°C and subsequently centrifuged for 20 min at 26,500 × *g* at 4°C. The pellets were dissolved in 100 µl buffer (50 mM HEPES, pH 7.4, 2 mM EDTA) via sonication. All buffers were supplemented with protease inhibitor (cOmplete EDTA-free, Roche) and phosphatase inhibitor (PhosSTOP, Roche). Protein quantification was carried out using the BCA protein assay kit from Biorbyt, according to the manufacturer's instructions.

### Proteomics

Short GeLC-Sequential Windowed data-independent Acquisition of the Total High-resolution Mass Spectra (SWATH-MS) was performed as previously described (Rodrigues et al., 2022). Briefly, 40 µg of each sample and a pooled sample per group were subjected to in-gel digestion after a partial SDS–PAGE run. LC–MS information was acquired in two different acquisition modes: data-dependent acquisition of the pooled samples and SWATH-MS acquisition of each individual sample. Protein identification and library construction were performed using ProteinPilot™ (v5.0.1, SCIEX), and the relative quantification was performed using the SWATH™ processing plug-in for PeakView™ (v2.2, SCIEX). The mass spectrometry proteomic data have been deposited to the ProteomeXchange Consortium via the PRIDE (Perez-Riverol et al., 2022) partner repository with the dataset identifier PXD047291.

### Whole-cell patch-clamp recordings

Acute striatal coronal slices (300 µM) were prepared from control and CS mice, as previously described (Rodrigues et al., 2022; Rodrigues and Monteiro, 2023; Santa et al., 2023). Briefly, mice were anaesthetized with avertin (tribromoethanol; 20 mg/ml; Sigma-Aldrich) with a dose of 0.5 mg/g body weight by intraperitoneal injection and transcardially perfused with *N*-methyl-d-glucamine-based artificial cerebrospinal fluid (NMDG-aCSF) solution as follows (in mM): 92 NMDG, 2.5 KCl, 1.2 NaH_2_PO_4_, 30 NaHCO_3_, 20 HEPES, 25 glucose, 5 sodium ascorbate, 2 thiourea, 3 sodium pyruvate, 10 MgSO_4_.7H_2_O, and 0.5 CaCl_2_.2H_2_O saturated with 95% O_2_ and 5% CO_2_, pH 7.2–7.4, 300–310 mOsm/L. Slices were cut using a vibratome (Leica Microsystems, VT1000S) and then incubated at 32–34°C for 11 min in a carbogenated NMDG-aCSF solution, followed by at least 1 h recovery at RT in a holding chamber (Brain Slice Keeper 4-Quad, AutoMate Scientific) filled with aCSF solution as follows (in mM): 119 NaCl, 2.5 KCl, 1.2 NaH_2_PO_4_, 24 NaHCO_3_, 12.5 glucose, 2 MgSO_4_.7H_2_O, and 2 CaCl_2_.2H_2_O saturated with 95% O_2_ and 5% CO_2_, pH 7.2–7.4, 300–310 mOsm/L.

Whole-cell patch-clamp recordings were performed using borosilicate glass pipettes (GB150F-8P, Science Products) pulled on a P1000 horizontal puller (Sutter Instrument) with resistances of ∼2–5 MΩ. Pipettes were filled with a cesium gluconate-based internal solution containing the following (in mM): 110 CsOH, 110 d-gluconic acid, 15 KCl, 4 NaCl, 5 TEA-Cl, 20 HEPES, 0.2 EGTA, 5 lidocaine *N*-ethyl chloride, 4 MgATP, and 0.3 Na_2_GTP, pH adjusted to 7.3 with KOH and osmolarity adjusted to 300 mOsm/L with K_2_SO_4_. To determine the *I*/*E* ratio of cortical neurons, glutamatergic and GABAergic synaptic transmission was assessed in the same cell by changing the membrane cell holding from −70 to 0 mV. The *I*/*E* ratio was calculated by determining the ratio of spontaneous inhibitory or excitatory postsynaptic current (sIPSC to sEPSC) frequency in each cell. Excitatory synaptic drive defined as sEPSC frequency × sEPSC amplitude and inhibitory synaptic drive defined as sIPSC frequency × sIPSC amplitude were measured in each sampled neuron. Signals were low-pass filtered at 2 KHz and digitized at 10 KHz using a Digidata 1440A. The recordings were made with a microelectrode amplifier in the voltage-clamp mode of operation (MultiClamp 700B, Molecular Devices). All recordings were performed under a BX-51WI microscope (Olympus), equipped with fluorescence and infrared differential interference contrast. Data was offline analyzed using Minianalysis software (Synaptosoft), and only cells with series-resistance values <25 MΩ were used.

### Statistical analysis

All statistical analyses were performed using Prism (GraphPad Software). Data were expressed as mean ± SEM. Significance was determined at the level of *p* < 0.05.

## Results

### Neuroproteomic analysis unveils altered glutamatergic and GABAergic synaptic composition in the mPFC of chronically stressed mice

**Figure 1.**
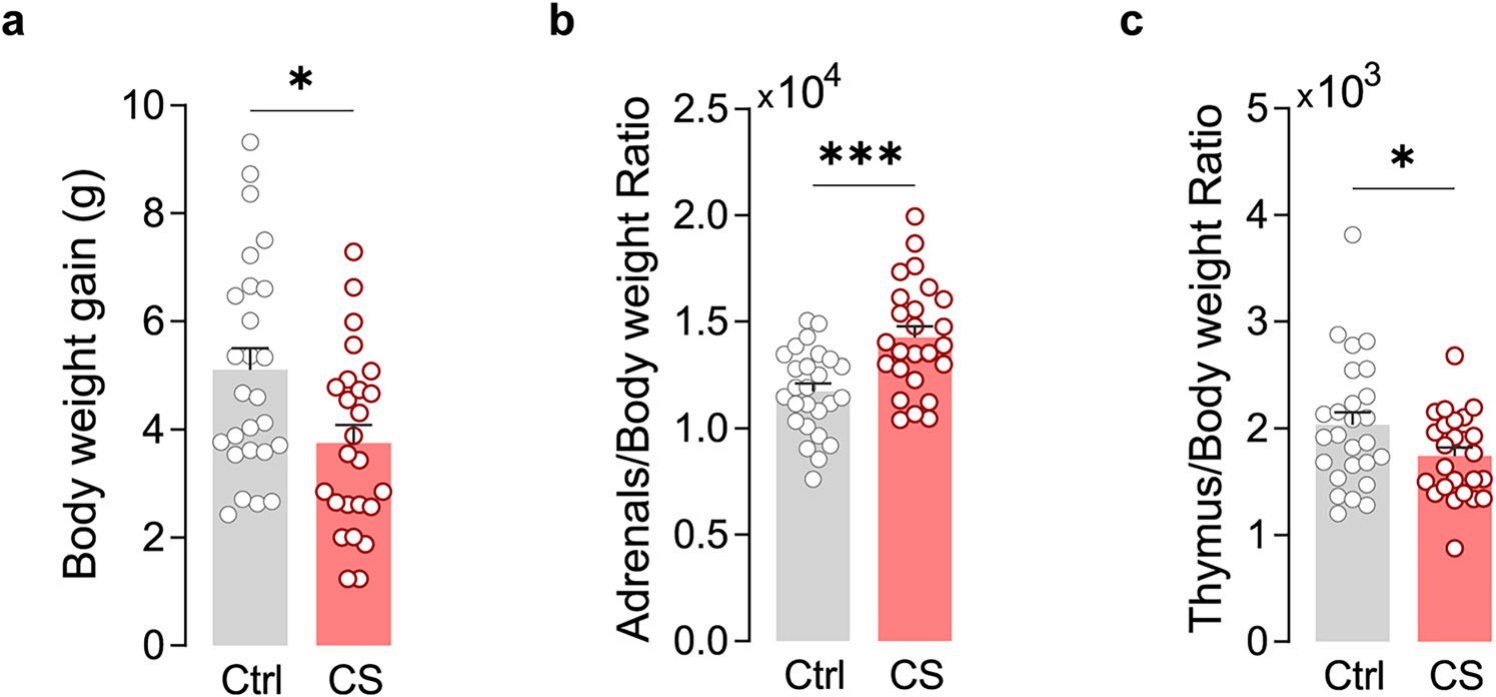
CS induces physiological alterations consistent with HPA axis activation. ***a***, Summary bar graphs [control (Ctrl) *N* = 26 and stressed (CS) *N* = 25 mice; *p = 0.0108] reveal a reduction in the body weight gain in CS mice. ***b***, Summary bar graphs (Ctrl *N* = 26 and CS *N* = 25 mice; ***p = 0.0003) of the *postmortem* adrenal gland weight over total body weight revealed that CS induces adrenal hypertrophy. ***c***, Summary bar graphs (Ctrl *N* = 26 and CS *N* = 25 mice; *p = 0.0462) of *postmortem* thymus weight over total body weight revealed that CS induces thymus atrophy. All bar graphs are mean ± SEM. Two-sided Welch's unpaired *t* test (***a–c***).

To investigate the impact of CS on mPFC circuits, we started by performing proteomic analysis of prefrontal samples obtained from both control and stressed mice. For that purpose, 5-week-old male mice assigned to the CS group underwent exposure to a well-established paradigm (Rodrigues et al., 2022). This paradigm involved three different stressors (forced swimming, immobilization, and social defeat) combined in an unpredictable manner and strategically designed to minimize the potential resilient effect stemming from behavioral control over the stressors (Amat et al., 2005). Following 21 d of daily stressor exposure, we assessed the overall physiological effect of CS. Comparative analysis with control littermates revealed that CS-exposed mice exhibited alterations indicative of prolonged hypothalamus–pituitary–adrenal (HPA) axis activation (de Kloet et al., 2005; Lupien et al., 2009; Ulrich-Lai and Herman, 2009). These alterations included reduced body weight gain (Fig. 1*a*), increased adrenal gland weight (Fig. 1*b*), and decreased thymus weight (Fig. 1*c*).

**Figure 2.**
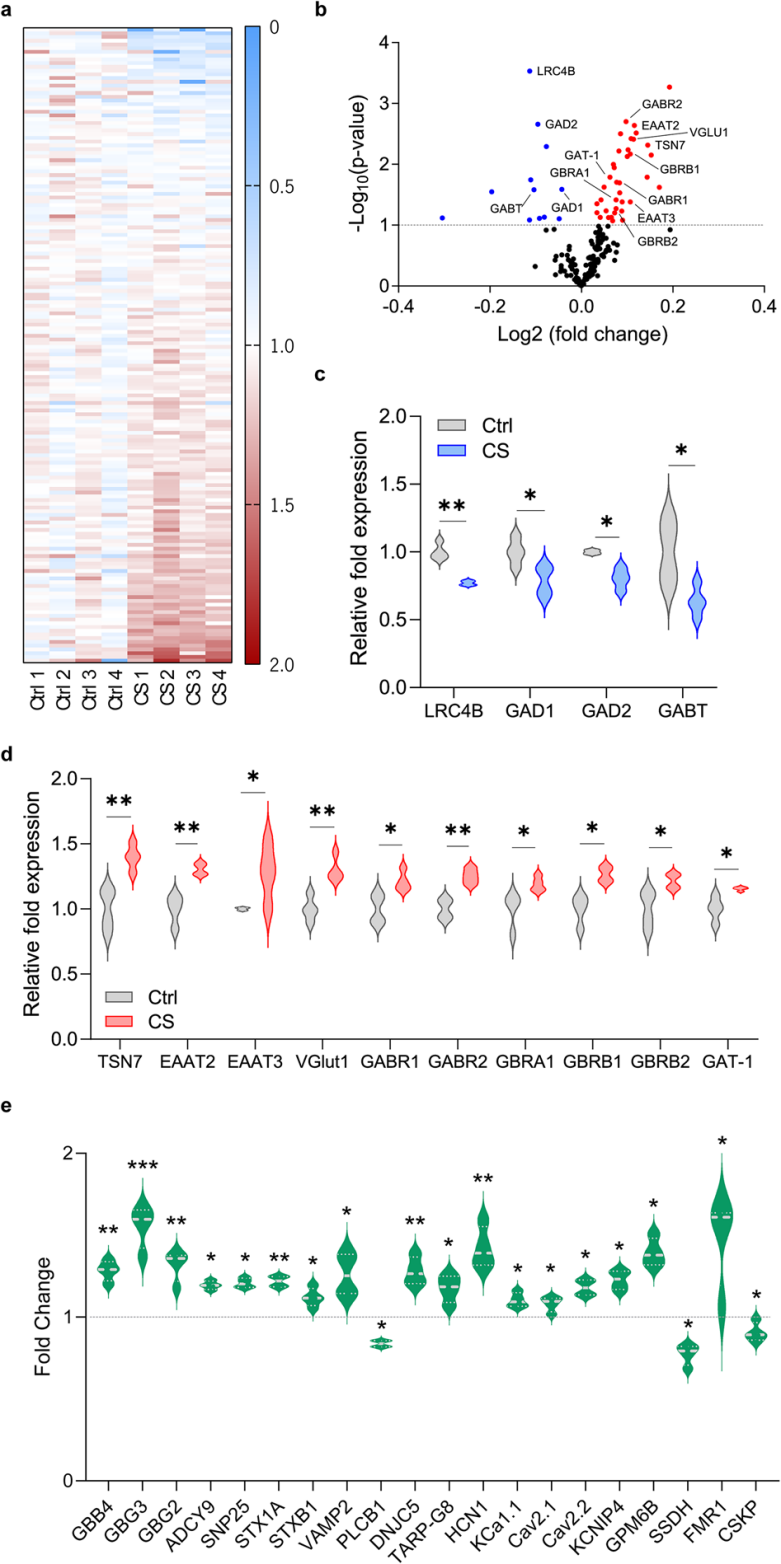
Proteomic analysis points to an *I*/*E* dysregulation caused by CS. ***a***, Heatmap view of 170 proteins (*y*-axis) from the “neuronal system�? family according to the Reactome database. Protein fold enrichment is color coded relative to the Ctrl average (blue, decreased expression; red, increased expression). *X*-axis represents biological replicates (Ctrl, 1–4; CS, 1–4). ***b***, Volcano plot visualization of proteins according to their significance (*p*-value) and fold change between Ctrl and CS mice, highlighting several significantly deregulated proteins of interest. Proteins significantly upregulated are represented in red, while downregulated are represented in blue. Proteins related to GABAergic and glutamatergic synapses are labeled. ***c***, Violin plot of GABAergic and glutamatergic synaptic proteins that are significantly downregulated. ***d***, Violin plot of GABAergic and glutamatergic synaptic proteins that are significantly upregulated. ***e***, Violin plot of proteins identified as belonging to the neuronal system pathway whose expression is significantly altered in CS mice. Each biological sample replicate is a combination of three brains pooled together (Ctrl *n* = 4 and CS *n* = 4 samples; *N* = 12 Ctrl and *N* = 12 CS mice). Two-sided Welch's unpaired *t* test (***c–e***) *p < 0.05, **p < 0.01, ***p < 0.001.

Shotgun proteomic analysis of prefrontal samples from stressed mice and their control littermates allowed the identification and quantification of 2,362 cortical-expressed proteins. Subsequent genome-wide overview and pathway analysis using the Reactome database (Croft et al., 2011; Gillespie et al., 2022) unveiled that among the identified proteins, 192 proteins were over-represented in neuronal systems' pathways. Downstream analyses were then performed on proteins with a coefficient of variation <30%, resulting in a narrowed sample size of 170 proteins (Fig. 2*a*). Volcano plot representation (Fig. 2*b*) allowed the visualization of a subset of neuronal proteins markedly changed between CS and control groups. Notably, from the 34 neuronal proteins identified as significantly altered (Fig. 2*c–e*), 9 were related to GABAergic synaptic transmission, while 5 were associated with the glutamatergic system (Fig. 2*c,d*). Specifically, we observed downregulation of three proteins involved in GABA metabolism (4-aminobutyrate aminotransferase, GABT; glutamate decarboxylase 1, GAD1, also known as GAD67 or DCE1; and glutamate decarboxylase 2, GAD2, also known as GAD65 or DCE2). Additionally, there were five upregulated GABA receptor subunits (GABA type B receptor subunits 1 and 2, GABR1 and GABR2, and GABA receptor subunits α1, β1, and β2, GBRA1, GBRB1, and GBRB2), along with one upregulated GABA transporter (GAT-1). Regarding the composition of excitatory synapses, our data revealed the downregulation of leucine-rich repeat-containing protein 4B, a regulator of excitatory synapse formation. Conversely, three glutamate transporters (excitatory amino acid transporter 2 and 3, EAAT2 and EAAT3, and type I vesicular glutamate transporter, VGlut1) exhibited upregulation, along with a protein involved in GluA2 receptor internalization (tetraspanin-7, TSN7). Collectively, our proteomic findings unveil several substantial alterations in both glutamatergic and GABAergic synaptic compositions following exposure to CS, indicating a potential functional *I*/*E* imbalance in prefrontal circuits.

### Layer 5/6 PNs, but not PV interneurons, from CS mice, show increased *I*/*E* ratio in both PL and IL cortices

**Figure 3.**
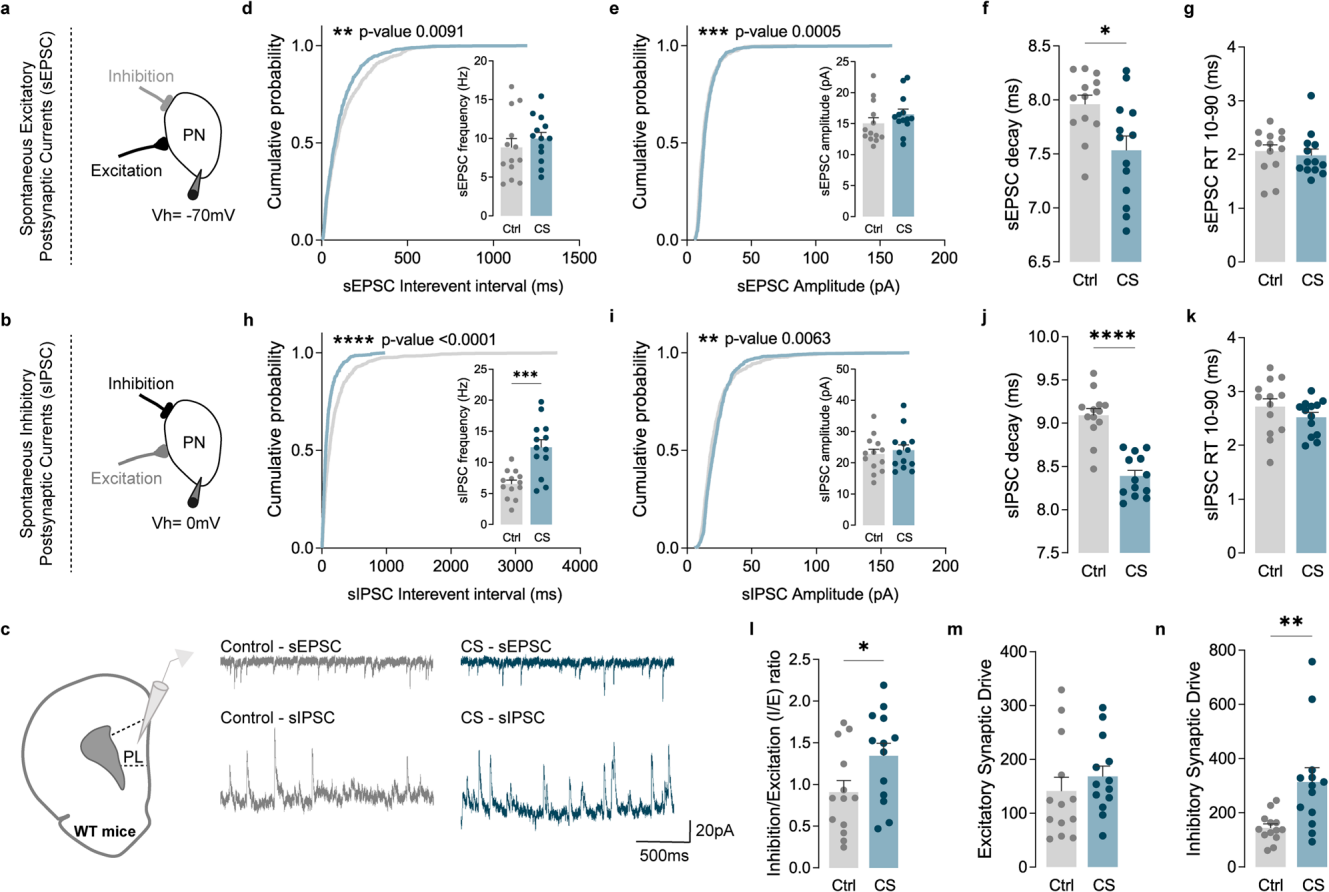
CS increases the *I*/*E* ratio in PL PNs by increasing inhibitory synaptic drive. ***a–c***, Protocol illustration of patch-clamp recordings obtained from L5/6 PNs in the PL subregion, in Ctrl (gray) and CS (blue) mice, with representative sEPSC and sIPSC traces. ***d***, Cumulative probability curves (50 events per cell; **p = 0.0091) and summary bar graphs (inset; Ctrl *n* = 13 and CS *n* = 13 cells from 3 Ctrl and 3 CS mice) show a left-shifted curve of sEPSC interevent interval in PNs of stressed mice, without affecting the average frequency of sEPSC. ***e***, Cumulative probability curves (50 events per cell; ***p = 0.0005) and summary bar graphs (inset; Ctrl *n* = 13 and CS *n* = 13 cells from 3 Ctrl and 3 CS mice) of sEPSC amplitude in PNs. ***f***, ***g***, Summary bar graphs (Ctrl *n* = 13 and CS *n* = 13 cells from 3 Ctrl and 3 CS mice) show significantly faster sEPSC decay kinetics (*p = 0.0126) in PNs of stressed mice, without changes in 10–90% RT. ***h***, Cumulative probability curves (50 events per cell; ****p < 0.0001) and summary bar graphs (inset; Ctrl *n* = 13 and CS *n* = 13 cells from 3 Ctrl and 3 CS mice; ***p = 0.0005) show an increase of sIPSC frequency in PNs of stressed mice. ***i***, Cumulative probability curves (50 events per cell; **p = 0.0063) and summary bar graphs (inset; Ctrl *n* = 13 and CS *n* = 13 cells from 3 Ctrl and 3 CS mice) of sIPSC amplitude in PNs. ***j***, ***k***, Summary bar graphs (Ctrl *n* = 13 and CS *n* = 13 cells from 3 Ctrl and 3 CS mice) show significantly faster sIPSC decay kinetics (****p < 0.0001) in PNs of stressed mice, without changes in 10–90% RT. ***l***, sIPSC/sEPSC (*I*/*E*) frequency ratio (Ctrl *n* = 13 and CS *n* = 13 cells from 3 Ctrl and 3 CS mice; *p = 0.0490) reveals an increase in *I*/*E* ratio in PNs of stressed mice. ***m***, Excitatory synaptic drive (Ctrl *n* = 13 and CS *n* = 13 cells from 3 Ctrl and 3 CS mice) reveals no differences between Ctrl and CS mice. ***n***, Inhibitory synaptic drive (Ctrl *n* = 13 and CS *n* = 13 cells from 3 Ctrl and 3 CS mice) reveals a remarkable increase after CS exposure. All bar graphs are mean ± SEM. For each box and whisker plot, the interior line shows the median, and the edges of the box are estimates of the first and third quartiles. The whiskers extend to the most extreme data points. Two-sided Welch's unpaired *t* test (***d–n***) and Kolmogorov–Smirnov test (***d***, ***e***, ***h–i*** curves).

**Figure 4.**
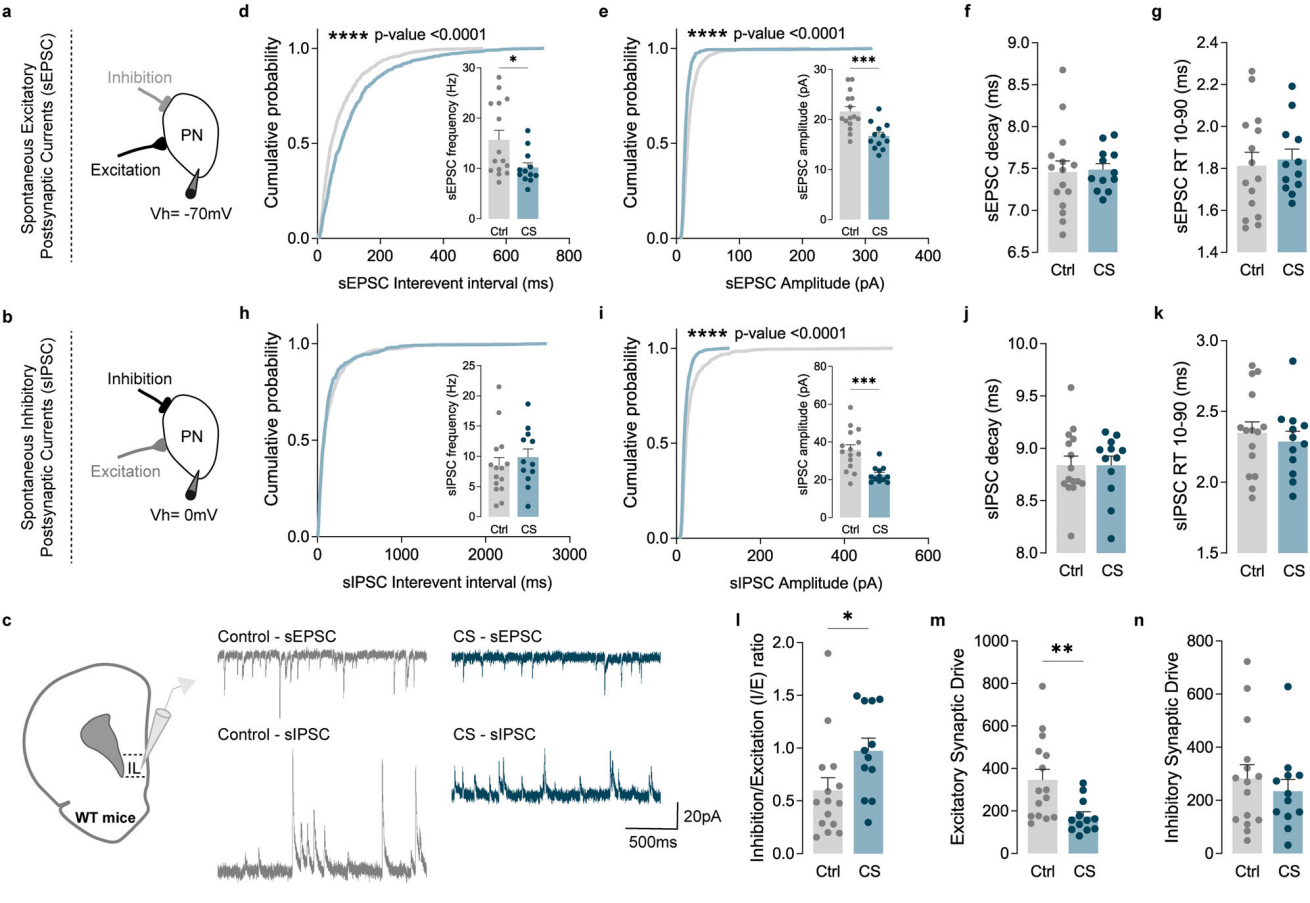
CS increases the *I*/*E* ratio in IL PNs by decreasing the excitatory synaptic drive. ***a–c***, Protocol illustration of patch-clamp recordings obtained from L5/6 PNs in the IL subregion, in Ctrl (gray) and CS (blue) mice and respective sEPSC and sIPSC representative traces. ***d***, Cumulative probability curves (50 events per cell; ****p < 0.0001) and summary bar graphs (inset; Ctrl *n* = 15 and CS *n* = 12 cells from 3 Ctrl and 3 CS mice; *p = 0.0170) show a significant decrease of sEPSC frequency in PNs of stressed mice. ***e***, Cumulative probability curves (50 events per cell; ****p < 0.0001) and summary bar graphs (inset; Ctrl *n* = 15 and CS *n* = 12 cells from 3 Ctrl and 3 CS mice; ***p = 0.0006) show a reduction of sEPSC amplitude in PNs of stressed mice. ***f***, ***g***, Summary bar graphs (Ctrl *n* = 15 and CS *n* = 12 cells from 3 Ctrl and 3 CS mice) show similar sEPSC decay kinetics and 10–90% RT in Ctrl and CS littermates. ***h***, Cumulative probability curves (50 events per cell) and summary bar graphs (inset; Ctrl *n* = 15 and CS *n* = 12 cells from 3 Ctrl and 3 CS mice) show no differences in sIPSC frequency in PNs of stressed mice. ***i***, Cumulative probability curves (50 events per cell; ****p < 0.0001) and summary bar graphs (inset; Ctrl *n* = 15 and CS *n* = 12 cells from 3 Ctrl and 3 CS mice; ***p = 0.0004) show reduced sIPSC amplitude in PNs of stressed mice. ***j***, ***k***, Summary bar graphs (Ctrl *n* = 15 and CS *n* = 12 cells from 3 Ctrl and 3 CS mice) show similar sIPSCs decay kinetics and 10–90% RT between PN from Ctrl and CS littermates. ***l***, sIPSC/sEPSC (*I*/*E*) frequency ratio (**p = 0.0071; Ctrl *n* = 15 and CS *n* = 12 cells from 3 Ctrl and 3 CS mice; **p = 0.0040) reveals an increase of *I*/*E* ratio in PNs of stressed mice. ***m***, Excitatory synaptic drive (Ctrl *n* = 15 and CS *n* = 12 cells from 3 Ctrl and 3 CS mice) shows a reduction in stressed mice. ***n***, Inhibitory synaptic drive (Ctrl *n* = 15 and CS *n* = 12 cells from 3 Ctrl and 3 CS mice) reveals no differences between Ctrl and CS mice. All bar graphs are mean ± SEM. For each box and whisker plot, the interior line shows the median, and the edges of the box are estimates of the first and third quartiles. The whiskers extend to the most extreme data points. Two-sided Welch's unpaired *t* test (***d–n***) and Kolmogorov–Smirnov test (***d***, ***e***, ***h–i*** curves).

**Figure 5.**
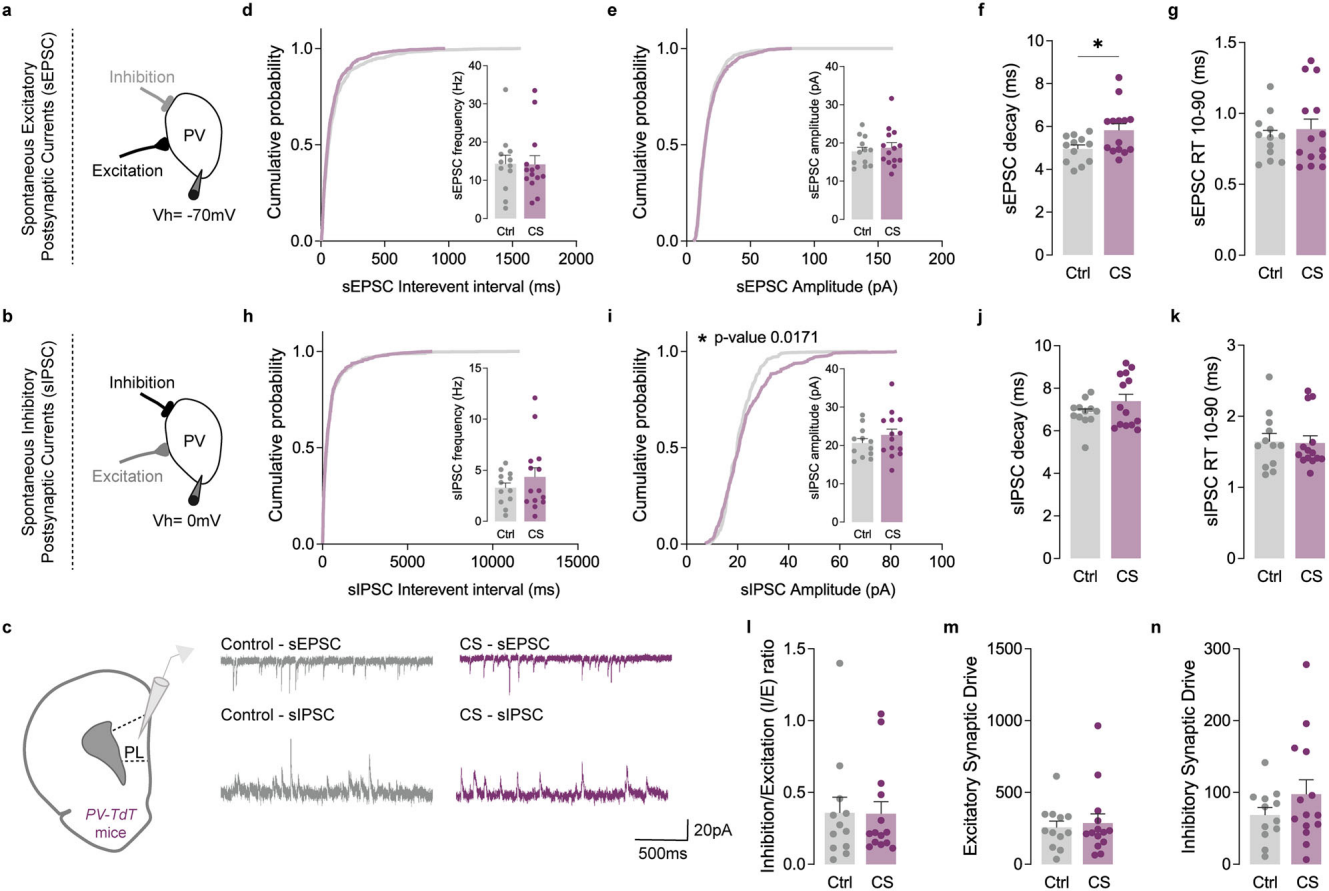
CS did not affect the *I*/*E* ratio of PV interneurons in the PL cortex. ***a–c***, Protocol illustration of patch-clamp recordings obtained from L5/6 fluorescently labeled PV interneurons in the PL subregion, in Ctrl (gray) and CS (purple) mice and respective sEPSC and sIPSC representative traces. ***d***, Cumulative probability curves (50 events per cell) and summary bar graphs (inset; Ctrl *n* = 12 and CS *n* = 14 cells from 3 Ctrl and 3 CS mice) show no alterations of sEPSC frequency in PV interneurons. ***e***, Cumulative probability curves (50 events per cell) and summary bar graphs (inset; Ctrl *n* = 12 and CS *n* = 14 cells from 3 Ctrl and 3 CS mice) show similar sEPSC amplitude in PV interneurons of Ctrl and CS mice. ***f***, ***g***, Summary bar graphs (Ctrl *n* = 12 and CS *n* = 14 cells from 3 Ctrl and 3 CS mice) show a slower sEPSC decay kinetics (*p = 0.0242) in PV interneurons of stressed mice, without alterations in the 10–90% RT. ***h***, Cumulative probability curves (25 events per cell) and summary bar graphs (inset; Ctrl *n* = 12 and CS *n* = 14 cells from 3 Ctrl and 3 CS mice) show no differences in sIPSC frequency in PV interneurons. ***i***, Cumulative probability curves (25 events per cell; *p = 0.0171) and summary bar graphs (inset; Ctrl *n* = 12 and CS *n* = 14 cells from 3 Ctrl and 3 CS mice) show a right-shifted curve of sIPSC amplitude in PV interneurons of CS mice, without affecting average sIPSC amplitude. ***j***, ***k***, Summary bar graphs (Ctrl *n* = 12 and CS *n* = 14 cells from 3 Ctrl and 3 CS mice) show similar sIPSC decay kinetics and 10–90% RT between PV interneurons from Ctrl and CS littermates. ***l***, sIPSC/sEPSC (*I*/*E*) frequency ratio (Ctrl *n* = 12 and CS *n* = 14 cells from 3 Ctrl and 3 CS mice) reveals no alterations in stressed mice. ***m***, Excitatory synaptic drive (Ctrl *n* = 12 and CS *n* = 14 cells from 3 Ctrl and 3 CS mice) reveals no differences between Ctrl and CS mice. ***n***, Inhibitory synaptic drive (Ctrl *n* = 12 and CS *n* = 14 cells from 3 Ctrl and 3 CS mice) reveals no differences between Ctrl and CS mice. All bar graphs are mean ± SEM. For each box and whisker plot, the interior line shows the median, and the edges of the box are estimates of the first and third quartiles. The whiskers extend to the most extreme data points. Two-sided Welch's unpaired *t* test (***d–n***) and Kolmogorov–Smirnov test (***d***, ***e***, ***h–i*** curves).

**Figure 6.**
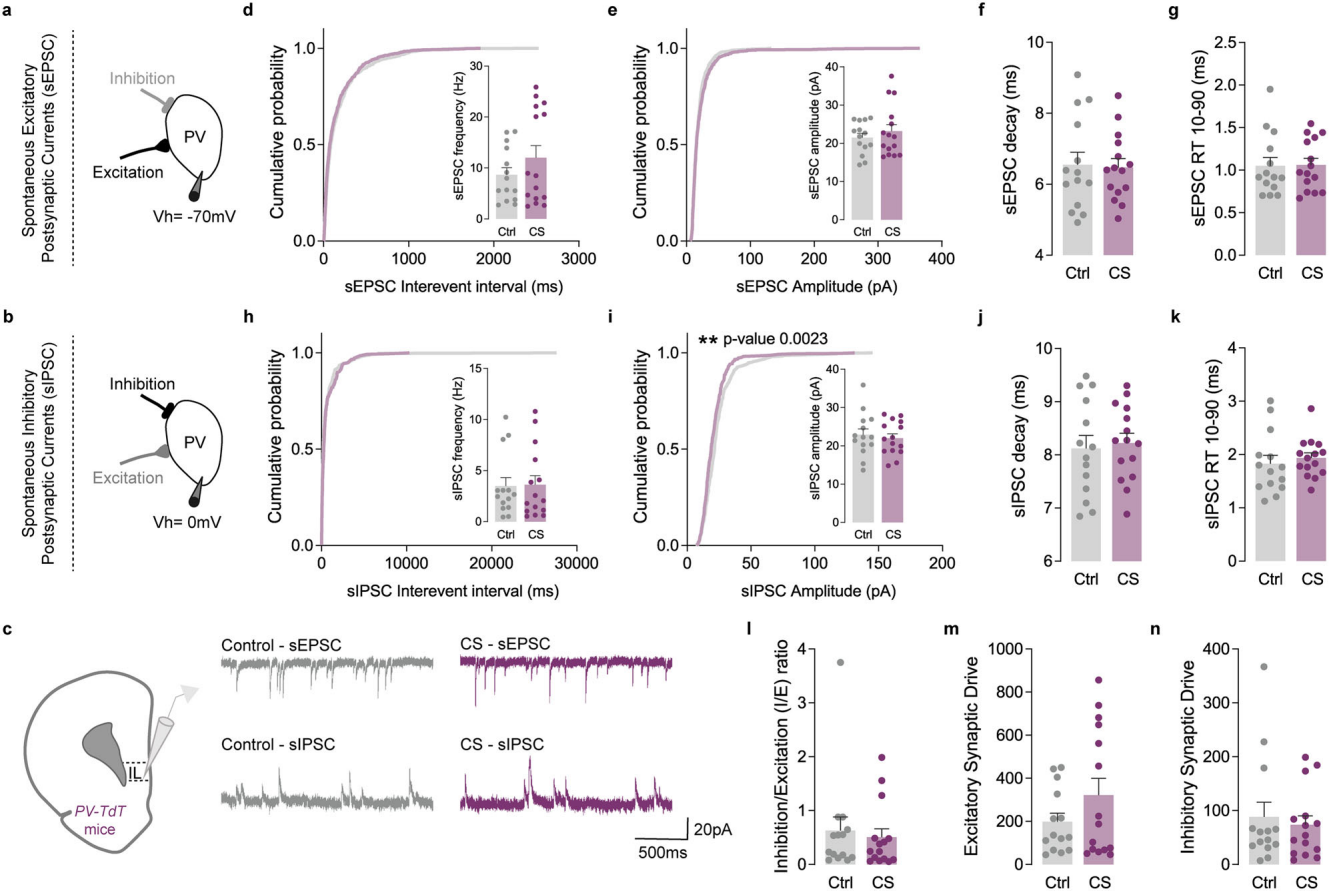
CS did not affect the *I*/*E* ratio of PV interneurons in the IL cortex. ***a–c***, Protocol illustration of patch-clamp recordings obtained from L5/6 fluorescently labeled PV interneurons in the IL subregion, in Ctrl (gray) and CS (purple) mice and respective sEPSC and sIPSC representative traces. ***d***, Cumulative probability curves (50 events per cell) and summary bar graphs (inset; Ctrl *n* = 14 and CS *n* = 15 cells from 3 Ctrl and 3 CS mice) show no alterations of sEPSC frequency in PV interneurons. ***e***, Cumulative probability curves (50 events per cell) and summary bar graphs (inset; Ctrl *n* = 14 and CS *n* = 15 cells from 3 Ctrl and 3 CS mice) show similar sEPSC amplitude in PV interneurons of CS mice. ***f***, ***g***, Summary bar graphs (Ctrl *n* = 14 and CS *n* = 15 cells from 3 Ctrl and 3 CS mice) show similar decay kinetics and 10–90% RT between PV interneurons from Ctrl and CS littermates. ***h***, Cumulative probability curves (25 events per cell) and summary bar graphs (inset; Ctrl *n* = 14 and CS *n* = 15 cells from 3 Ctrl and 3 CS mice) show no differences in sIPSC frequency in PV interneurons. ***i***, Cumulative probability curves (25 events per cell; **p = 0.0023) and summary bar graphs (inset; Ctrl *n* = 14 and CS *n* = 15 cells from 3 Ctrl and 3 CS mice) show a left-shifted curve of sIPSC amplitude in PV interneurons of CS mice, without affecting average sIPSC amplitude. ***j***, ***k***, Summary bar graphs (Ctrl *n* = 14 and CS *n* = 15 cells from 3 Ctrl and 3 CS mice) show similar sIPSC decay kinetics and 10–90% RT between PV interneurons from Ctrl and CS littermates. ***L***, sIPSC/sEPSC (*I*/*E*) frequency ratio (Ctrl *n* = 14 and CS *n* = 15 cells from 3 Ctrl and 3 CS mice) reveals no alterations in CS mice. ***m***, Excitatory synaptic drive (Ctrl *n* = 14 and CS *n* = 15 cells from 3 Ctrl and 3 CS mice) reveals no differences between Ctrl and CS mice. ***n***, Inhibitory synaptic drive (Ctrl *n* = 12 and CS *n* = 14 cells from 3 Ctrl and 3 CS mice) reveals no differences between Ctrl and CS mice. All bar graphs are mean ± SEM. For each box and whisker plot, the interior line shows the median, and the edges of the box are estimates of the first and third quartiles. The whiskers extend to the most extreme data points. Two-sided Welch's unpaired *t* test (***d–n***) and Kolmogorov–Smirnov test (***d***, ***e***, ***h–i*** curves).

The balance between synaptic excitation and inhibition plays a pivotal role in regulating information flow within the brain. Disruptions in this balance, known as *I*/*E* ratio imbalances, have been implicated in several stress-related disorders and suggested as a consequence of CS exposure (Luscher et al., 2011; Ferguson and Gao, 2018; Selten et al., 2018; Page and Coutellier, 2019). Our neuroproteomic data indeed suggest a stress-induced *I*/*E* imbalance, potentially serving as a mechanism through which CS may impair cortical function. Earlier studies have demonstrated that stress induces dendritic remodeling in both PL and IL PNs (Dias-Ferreira et al., 2009), contributing to abnormal action selection strategies (Dias-Ferreira et al., 2009). Additionally, prior research revealed that CS induces functional changes in Layer 5/6 (L5/6) cortical neurons (Rodrigues and Monteiro, 2023). Building upon this collective knowledge and integrating it with the data obtained from proteomics, we sought to investigate whether CS impacts the synaptic *I*/*E* ratio in PL and IL cortices. To address this question, we conducted recordings of sEPSC and sIPSC, respectively. Leveraging the distinct reversal potentials for AMPA-receptor-mediated EPSC (0 mV) and GABA receptor-mediated IPSC (−70 mV), we employed selective voltage-clamp recordings to isolate sEPSCs and sIPSCs (Figs. 3*a–c*, 4*a–c*, 5*a–c*, 6*a–c*).

Our data revealed that, compared with control littermates, PL PNs from stressed mice exhibited a left-shifted sEPSC curve of interevent interval, with a trend for a higher frequency average of spontaneous excitatory events (Fig. 3*d*). A trend for higher sEPSC amplitude also seems to be present in the CS group (Fig. 3*e*). These alterations were accompanied by reduced sEPSC decay kinetics, indicative of different subunit receptor compositions after CS exposure (Fig. 3*f*), without affecting the 10–90% rise time (RT; Fig. 3*g*). Additionally, these same cells from stressed mice presented an increase in sIPSC frequency (Fig. 3*h*) and reduced sIPSC decay kinetics (Fig. 3*j*), without significant changes in the average amplitude of sIPSC (Fig. 3*i*). No differences were observed in sIPSC 10–90% RT (Fig. 3*k*). These data demonstrate that, in the PL subregion, CS mainly enhances the frequency of spontaneous inhibitory transmission without affecting much excitatory transmission, causing an *I*/*E* dysregulation. Indeed, PL PNs revealed a significant increase in the *I*/*E* (Fig. 3*l*) caused by an increase in inhibitory synaptic drive (Fig. 3*n*). The excitatory synaptic drive of PL PNs was shown not to be altered by CS (Fig. 3*m*).

Next, we studied the impact of CS on IL PNs. Voltage-clamp recordings revealed markedly reduced sEPSC frequency (Fig. 4*d*) and amplitude (Fig. 4*e*) in CS mice and similar sEPSC decay kinetics and 10–90% RT (Fig. 4*f,g*). Furthermore, reduced sIPSC amplitude (Fig. 4*i*) was also observed in IL pyramidal cells of stressed mice, with no differences in frequency (Fig. 4*h*). No significant changes were observed in sIPSC decay kinetics and 10–90% RT (Fig. 4*j,k*). Accordingly, when compared with control littermates, IL PNs from stressed mice showed a marked increase in *I*/*E* ratio and excitatory synaptic drive (Fig. 4*l,m*), with no changes in the inhibitory synaptic drive (Fig. 4*n*).

Overall, our data demonstrated that CS increases *I*/*E* balance in both IL and PL PNs by different mechanisms: while PL PNs presented an increase in the inhibitory synaptic drive caused by increased frequency of spontaneous inhibitory transmission, IL PNs showed a decrease in the excitatory synaptic drive instigated by decreased frequency and amplitude of spontaneous excitatory transmission.

Although PNs serve as the exclusive output neurons of the mPFC, their activity is significantly influenced by local PV interneurons (Harris and Shepherd, 2015; Tremblay et al., 2016). Despite constituting only 2% of the mPFC neuronal population (Nahar et al., 2021), PV interneurons play a crucial role in modulating cortical circuits. They provide essential feedback and feedforward inhibition and contribute to generating rhythmic and synchronized network activity (McBain and Fisahn, 2001). Our data from PNs revealed a pronounced impairment in inhibitory transmission within the mPFC in stressed mice. Consequently, we investigated whether the E/I ratio could also be disrupted in PV interneurons following CS. Employing the same methodology, we conducted recordings of sEPSC and sIPSC from fluorescently labeled PV cells in the IL and PL cortices of Pvalb-tdTomato mice exposed to CS. Surprisingly, our analysis revealed no discernible changes in this population (Figs. 5, 6). This suggests that CS specifically induces a synaptic prefrontal *I*/*E* imbalance in PNs.

Altogether our data support the hypothesis that CS induces mPFC hypofunction, by increasing the *I*/*E* ratio and impairing the excitatory or inhibitory synaptic drive of PNs on both PL and IL subregions.

## Discussion

Imbalances in prefrontal cortical excitatory and inhibitory neurotransmission have been implicated in various neuropsychiatric disorders, including depression, bipolar disorder, and schizophrenia (Beasley et al., 2002; Sakai et al., 2008; Fung et al., 2010; Lisman, 2012; Selimbeyoglu et al., 2017), as well as in CS (Page and Coutellier, 2019). However, the precise nature of stress-induced *I*/*E* dysregulation and its role in triggering mental disorders remain unclear, with some studies presenting contradictory findings in glutamatergic and GABAergic systems (Yuen et al., 2012; McKlveen et al., 2016).

In our study, we investigated the functional changes induced by CS in the *I*/*E* balance of IL and PL cortices of male mice. Our findings highlight that CS specifically impacts L5/6 PNs, reducing the synaptic *I*/*E* ratio in both prefrontal cortical regions, without affecting PV interneurons. Moreover, we elucidated that the mechanisms driving this *I*/*E* imbalance differ between IL and PL PNs. Specifically, CS exposure increased the frequency of spontaneous GABAergic transmission onto PL PNs while simultaneously diminishing the frequency and amplitude of spontaneous glutamatergic transmission onto IL PNs. These differences result in dysfunctional excitatory and inhibitory synaptic drives in IL and PL neurons, respectively. Importantly, our results in mice confirm findings previously reported in studies using Sprague Dawley rats, indicating that CS affects synaptic transmission in mPFC, supporting the hypothesis that mPFC hypofunction may trigger mental illness and induce stress-related behavioral symptoms (Yuen et al., 2012; McKlveen et al., 2016). Notably, Yuen et al. (2012) reported decreased excitatory synaptic transmission in PNs from L5 of the prefrontal cortex, while McKlveen et al. (2016) showed a trend toward decreased excitatory synaptic transmission in IL PNs, coupled with increased inhibitory synaptic transmission, resulting in an increased *I*/*E* ratio. While these findings are in line with our data, it is important to note methodological differences. Yuen et al. (2012) and McKlveen et al. (2016) investigated synaptic transmission through recordings of miniature postsynaptic currents (mPSCs), while our study recorded spontaneous postsynaptic currents (sPSCs). This distinction is critical because mPSCs reflect postsynaptic responses to the quantal release of neurotransmitters from individual vesicles without presynaptic stimulation, serving more as a proxy for the number of synapses. In contrast, sPSCs capture postsynaptic currents resulting from spontaneous presynaptic activity, reflecting the overall synaptic drive and network activity impacting the neuron. By recording sEPSCs and sIPSCs in the same cell, we can assess the dynamic interplay between excitatory and inhibitory inputs, providing a more physiological understanding of synaptic integration and *I*/*E* ratio in the context of an intact neural circuit. Thus, our approach offers a comprehensive understanding of how CS alters synaptic transmission. Moreover, our results demonstrate that CS increases the *I*/*E* ratio in the mPFC of mice, corroborating findings by McKlveen et al. (2016) in rats, thus validating these findings across both species and highlighting the IL as a critical hub for stress-related disorders.

CS has been associated with impairments in decision-making and the potentiation of habit formation (Dias-Ferreira et al., 2009; Friedman et al., 2017), thereby hindering the expression of goal-directed behaviors (Dias-Ferreira et al., 2009; Friedman et al., 2017). In rodents, the mPFC is recognized for mediating goal-directed and habitual behaviors through the PL and IL cortices, respectively (Amaya and Smith, 2018; Smith and Laiks, 2018). In line with previous research, the observed enhancement of GABAergic transmission and increased inhibitory synaptic drive onto PL PNs in CS-exposed mice may contribute to functional impairments in this brain region and downstream limbic structures responsible for regulating such behaviors. This, in turn, could result in diminished goal-directed acquisition and/or expression, aligning with the well-documented impact of CS on decision-making processes.

Our neuroproteomic analysis unveiled alterations in five proteins associated with excitatory synaptic transmission following exposure to CS. Specifically, we observed a downregulation of LCR4B, also known as Netrin-G ligand-3. LCR4B is a postsynaptic adhesion molecule known to regulate excitatory synapse formation and function through direct interactions with the PSD-95 and the presynaptic LAR family receptor tyrosine phosphatases (S. Kim et al., 2006; Woo et al., 2009a,b). Consistent with our electrophysiological findings, prior studies have demonstrated that the knockdown of LCR4B protein leads to a reduction in excitatory synapse number and function (Woo et al., 2009a). Furthermore, we noted an upregulation of VGlut1, EAAT2, EAAT3, and TSN7 proteins. VGlut1 is involved in transporting glutamate to synaptic vesicles on presynaptic neurons, influencing glutamate release. EAAT2 and EAAT3 are responsible for the reuptake of this neurotransmitter from the synaptic cleft. Additionally, the upregulation of TSN7 may suggest fewer glutamate receptor 2 (GluA2) molecules present on the postsynaptic membrane of neurons. The collective overexpression of these proteins suggests an overall decrease in the availability of glutamate at the synaptic cleft and a reduction in GluA2 at the synaptic surface. These changes may contribute to the observed deficits in glutamatergic excitatory synaptic transmission providing mechanistic insights into the functional alterations induced by CS.

The interpretation of the inhibitory synaptic proteins identified by proteomics becomes more intricate, given that we observed increased frequency of sIPSC in PL PNs but reduced amplitude of sIPSC in IL PNs. However, it is noteworthy to highlight the upregulation of the hyperpolarization-activated cyclic nucleotide-gated channel 1 (HCN1; Fig. 2*b*), an ion channel associated with stress-related disorders, including schizophrenia (Chen et al., 2022) and depression (Shah, 2012; C. S. Kim and Johnston, 2018). HCN1 has already been linked to stress-induced depressive behaviors (C. S. Kim et al., 2018). This ion channel assumes particular significance as its expression on presynaptic GABAergic terminals has been shown to limit GABAergic transmission onto L5/6 PNs in the mPFC (C. S. Kim et al., 2018). The overexpression of HCN1 in GABAergic terminals might be a possible mechanism by which CS shapes inhibitory transmission in the mPFC. Although we did not detect significant changes in the *I*/*E* ratio of cortical PV interneurons, we should emphasize that our methodology did not allow for the differentiation of existing cortical PV interneuron subtypes. Consequently, we cannot exclude the possibility that CS may selectively shape the *I*/*E* ratio of a specific subtype of PV interneurons.

Despite the relevance of our data, we must highlight that our study was carried out exclusively on male mice and therefore our conclusions can be attributed to male mice only. The vulnerability to CS and the neuronal and behavioral responses to stress are known to be sex dependent (Kessler and McLeod, 1984; Kudielka and Kirschbaum, 2005; Bale, 2006; Gruene et al., 2015; Hodes et al., 2015; Furman et al., 2022) and highly influenced by the estrous cycle in females (Blume et al., 2019; Jaric et al., 2019). Moreover, in our study, we employed social defeat as a stressor, which is challenging to implement in females due to their lack of natural aggression and strong territoriality (Palanza and Parmigiani, 2017; Atrooz et al., 2021). Therefore, to avoid potential biological heterogeneity that could complicate the interpretation of our results, we chose to focus exclusively on males in this study. However, future studies should investigate the susceptibility of female prefrontal cortex circuits to CS.

Collectively, our study furnishes compelling evidence of a substantial shift in the *I*/*E* balance within IL and PL PNs resulting from CS. This nuanced understanding of the stress-induced alterations in *I*/*E* balance contributes valuable insights into the intricate mechanisms underpinning the link between CS and neuropsychiatric disorders.

## Synthesis

Reviewing Editor: Ayako Watabe, The Jikei University School of Medicine Decisions are customarily a result of the Reviewing Editor and the peer reviewers coming together and discussing their recommendations until a consensus is reached. When revisions are invited, a fact-based synthesis statement explaining their decision and outlining what is needed to prepare a revision will be listed below. The following reviewer(s) agreed to reveal their identity: Hiroshi Nomura.

The authors explore how chronic stress affects the balance between excitatory and inhibitory synaptic transmission in pyramidal neurons within the prefrontal cortex. Given the pivotal role of the prefrontal cortex in managing working memory and executive functions, and its link to various stress-related disorders, the study holds significant potential for elucidating the mechanisms underlying certain neuropsychiatric conditions. However, there are several immature points in the manuscripts, and it does not seem possible to move forward in the area of chronic stress and synaptic transmission in the PFC at this time.

1. Numerous pioneering studies, including two directly relevant to this manuscript, have examined how chronic stress influences synaptic transmission in the PFC. McKlveen et al (Biol Psychiatry, 2016) found that chronic stress enhances miniature IPSC frequency in infralimbic cortex neurons. Yuen et al. (Neuron, 2012) observed a reduction in excitatory inputs to the prelimbic cortex neurons. These studies, employing electrophysiology, molecular, and behavioral approaches, suggest chronic stress impacts the infralimbic and prelimbic cortex neurons differently, yet commonly disrupts the excitatory-inhibitory balance. These findings bring into question the novelty of the research question posed by the authors. It might be beneficial to clarify the differences in methods and results between the current and previous studies and, if necessary, consider additional experiments or analyses to refine the research focus.

2. The evidence presented to support the claim that chronic stress alters the excitatory/inhibitory (E/I) balance is not convincing. The authors' definition of E/I balance is vague. For example, among the four metrics related to E/I balance shown in Figure 2 (E/I ratio, log(E/I ratio), synaptic drive, log(synaptic drive)), only one (log(E/I ratio) demonstrated a significant difference. This outcome raises questions about the actual impact of chronic stress on E/I balance. The synaptic drive might be a more suitable measure for evaluating E/I balance in experiments that record sEPSCs and sIPSCs.

3. The link between proteomics and electrophysiology experiments is weak. While the electrophysiology experiments focus on comparisons between the infralimbic and prelimbic areas, and between excitatory neurons and parvalbumin-positive neurons, the proteomics analysis does not differentiate between cell types, making it difficult to discuss their relevance. Additionally, the lack of experimental manipulation of identified proteins complicates the discussion of their relationships.

4. The manuscript does not specify the number of mice used in the electrophysiology experiments.

5. It is not clear why only males were used in the study. Authors are invited to discuss whether sex may represent a biological variable.
